# Peeled Guidewire Coating with Debulked Plaque Obtained by Directional Coronary Atherectomy

**DOI:** 10.1155/2017/2397183

**Published:** 2017-04-05

**Authors:** Rikuta Hamaya, Taishi Yonetsu, Sadamitsu Ichijo, Makoto Araki, Tadashi Murai, Yoshihisa Kanaji, Eisuke Usui, Junji Matsuda, Masahiro Hoshino, Masahiro Hada, Takayuki Niida, Yoshinori Kanno, Tsunekazu Kakuta

**Affiliations:** Division of Cardiovascular Medicine, Tsuchiura Kyodo General Hospital, Ibaraki, Japan

## Abstract

Percutaneous directional coronary atherectomy (DCA) is a plaque debulking method performed in Japan, and recently a renewed DCA device has been launched. We present a case with a tight left anterior descending lesion undergoing percutaneous coronary intervention with application of DCA. After several sessions of DCA, white plaques accompanied by green, stringed materials were obtained from the device; some materials were considerably long (approximately 15 mm in length). A drug-eluting stent was subsequently implanted, and the procedure was completed successfully without any complications. The extracted plaques and artificial materials were pathologically examined, and no inflammatory changes were detected on plaques adjacent to the material. Assessing pathological findings and structure of the DCA catheter, the obtained artificial materials were considered as peeled guidewire, possibly resulting from the friction between the guidewire and metallic bearing in the housing of DCA catheter. Of note, this phenomenon has been recognized even in other DCA cases in which guidewires of the other kind are used. We report this phenomenon for the first time, warning of theoretically possible distal embolization of artificial materials caused by any debulking devices.

## 1. Introduction

Percutaneous directional coronary atherectomy (DCA) had been utilized dating back to late 1980s as an adjunctive procedure during percutaneous coronary intervention (PCI) for the reduction of plaque volume, especially for bifurcation or ostium lesions [1, 2]. However, the advent of coronary stents including drug-eluting stents overwhelmed the benefit of DCA. Moreover, given the technical expertise required for the DCA procedure which further reduced the clinical indication, DCA had gone out of use [3]. Nevertheless, a renewed DCA device has been recently launched in Japan, and potential benefits have been shown in the integrated strategy using DCA followed by stenting in selected populations [1]. We report a case treated with use of the renewed DCA device (Atherocut, NIPRO, Tokyo, Japan), in which artificial materials that potentially belonged to a guidewire coating were obtained as well as atheromatous plaque.

## 2. Case Presentation

**Figure 1:**
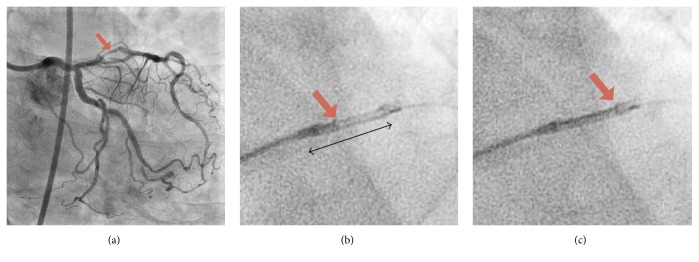
(a) Coronary angiogram showing a tight stenosis in proximal left anterior descending artery (culprit lesion shown by red arrow). (b and c) Directional coronary atherectomy (DCA) catheter debulking plaque of the target lesion. Red arrows show the head of DCA cutter, advancing from proximal (b) to distal (c) position of the catheter. Bracket indicates the housing area of the catheter.

**Figure 2:**
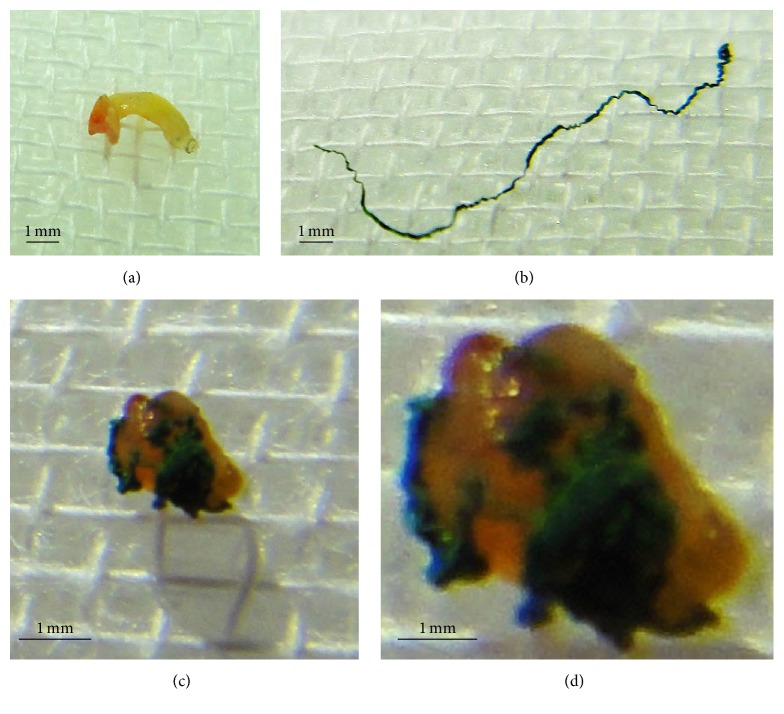
Directional coronary atherectomy-derived plaque (a) and artificial material (b), which was considered as peeled guidewire, approximately 15 mm in length. Some plaques were attached to the material (c and d [magnified image]).

**Figure 3:**
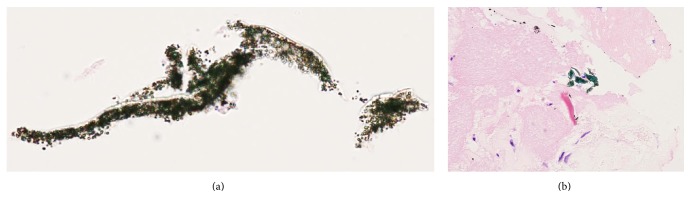
Pathological specimen of the peeled guidewire (a) and fragments of the material shown in the plaque (b). There are no inflammatory findings on plaque adjacent to the material.

A 73-year-old male with effort angina was referred to our hospital, and diagnostic coronary angiography revealed a tight stenosis in proximal left anterior descending artery (LAD) (Figure 1(a)), which demonstrated a fractional flow reserve (FFR) of 0.68. Given the positive FFR and clinical symptom, PCI was indicated. Intravascular ultrasound (IVUS) revealed an eccentric fibrous plaque with mild calcification predominantly to the direction to myocardium. With the use of a 300 cm guidewire (Gland Slam, ASAHI INTECC CO. Nagoya, Japan), we performed IVUS-guided DCA. Figures 1(b) and 1(c) show DCA catheter debulking targeted plaque. After 7 sessions of debulking, white plaques accompanied by green, stringed materials were obtained from the device (Figure 2). Some extracted artificial materials were considerably large, approximately 15 mm in length (Figure 2(b)); some were clumped and attached to plaques (Figures 2(c)-2(d)). The green, stringed, and tangled materials were speculated to be an artificial fragment of the guidewire coating to the eye balls. A drug-eluting stent was subsequently implanted with an optimal stent expansion, and this procedure was completed successfully without any complications. The extracted plaques and artificial materials were pathologically examined (Figure 3). Plaques were mainly fibrotic with partial hyalinization and myxoid changes. A few calcium deposits and cholesterol crystals were identified as well. There was no atheromatous change or intraluminal thrombus attached to the plaque. Green-colored materials consisted of cluster of fine granules, the colors of which were black in hematoxylin and eosin stain and blue by the polarizing microscope. Inflammatory changes were not detected within the plaque adjacent to the materials.

## 3. Discussion

To the best of our knowledge, this is the first report of peeled artificial materials obtained by the recently relaunched DCA device. DCA has potential advantage in reducing residual plaque volume with combined use of IVUS and the appropriate understanding, as well as in obtaining targeted plaque by tissue [4–6]. The complications of prior DCA device included coronary perforation, dissection, and deforming implanted stent [3, 7]; although DCA device has been renewed and the availability is limited to a specific country, this report is of potential importance for theoretically possible distal embolization of artificial materials caused by any debulking devices [8].

DCA device cut plaques off by a fast rotating cutter, at approximately 6000 rpm, which is pushed on the coronary plaques by inflated balloon equipped to the DCA catheter. The renewed DCA catheter, in order to hold the axis of the cutter to the center of the device, mounts “a guidewire-supporting bearing” at the distal end of the cutter housing. As this metallic bearing tightly holds the guidewire, the friction between guidewire and the bearing is produced during cutting, which may have resulted in a peeling of the wire surface. These hypotheses are supported by the pathological examination, which revealed that there were no inflammatory changes found in the attachment of plaques and the materials, implicating that this phenomenon occurred inside the DCA catheter. In our case, the target plaque, which was pathologically a fibrous tissue, was effectively reduced without clinical complication; however, the extracted artificial material could have caused significant myocardial damage by distal embolization, considering the size of the materials that was approximately 15 mm in length. Of note, at our institution, this phenomenon has been recognized even in other DCA cases in which guidewires of the other kind are used (FLEXI-wire, Abbott Vascular Japan, Tokyo, Japan; ABYSS DCA support 300, Nipro, Osaka, Japan). This phenomenon is not uncommon; in the recently performed consecutive 10 cases treated by using this DCA device, foreign artificial materials (presumably wire-coating debris) were recognized in four cases by eye balls.

Although the peeled materials have been confirmed inside the DCA devices in these cases, we could not cancel the possibility of distal embolization of these materials if they are prolapsed from the housing of debulking cutter. Given the specification of the device, the high rotational speed and tight wire bearing of the device, it might be difficult to have a complete solution to this phenomenon because wire coating is not likely to be tolerable to the friction. Nevertheless, potential improvement of wire coating or coaxial movement of the cutter along a guidewire may reduce this unfavorable phenomenon. Additionally, considering the possible mechanism of this phenomenon, the most efficient ways to prevent distal embolization would be to limit the number of cuts and to be cautious not to open the housing while the device is being withdrawn into the guiding catheter, keeping the nose-cone not filled up with obtained materials. Distal protection device could be an option in regard to minimization of the risk of distal embolization, yet the safety and effectiveness are not validated. Further studies are warranted to precisely investigate the mechanism of peeling and the adverse effects in clinical settings.
